# EZN-2208 (PEG-SN38) Overcomes ABCG2-Mediated Topotecan Resistance in BRCA1-Deficient Mouse Mammary Tumors

**DOI:** 10.1371/journal.pone.0045248

**Published:** 2012-09-17

**Authors:** Serge A. L. Zander, Wendy Sol, Lee Greenberger, Yixian Zhang, Olaf van Tellingen, Jos Jonkers, Piet Borst, Sven Rottenberg

**Affiliations:** 1 Division of Molecular Oncology, The Netherlands Cancer Institute, Amsterdam, The Netherlands; 2 Division of Molecular Pathology, The Netherlands Cancer Institute, Amsterdam, The Netherlands; 3 Department of Pharmacology, Enzon Pharmaceuticals Inc., Piscataway, New Jersey, United States of America; 4 Department of Clinical Chemistry, The Netherlands Cancer Institute, Amsterdam, The Netherlands; Indiana University School of Medicine, United States of America

## Abstract

BRCA1 dysfunction in hereditary breast cancer causes defective homology-directed DNA repair and sensitivity towards DNA damaging agents like the clinically used topoisomerase I inhibitors topotecan and irinotecan. Using our conditional *K14cre*;*Brca1^F/F^*;*p53^F/F^* mouse model, we showed previously that BRCA1;p53-deficient mammary tumors initially respond to topotecan, but frequently acquire resistance by overexpression of the efflux transporter ABCG2. Here, we tested the pegylated SN38 compound EZN-2208 as a novel approach to treat *BRCA1*-mutated tumors that express ABCG2. We found that EZN-2208 therapy resulted in more pronounced and durable responses of ABCG2-positive tumors than topotecan or irinotecan therapy. We also evaluated tumor-specific ABCG2 inhibition by Ko143 in *Abcg2^−/−^* host animals that carried tumors with topotecan-induced ABCG2 expression. Addition of Ko143 moderately increased overall survival of these animals, but did not yield tumor responses like those seen after EZN-2208 therapy. Our results suggest that pegylation of Top1 inhibitors may be a useful strategy to circumvent efflux transporter-mediated resistance and to improve their efficacy in the clinic.

## Introduction

Poisoning topoisomerase I (Top1) is a useful therapeutic approach to target cancer cells with intrinsic defects in the DNA damage response [1]. In particular the camptothecin derivatives topotecan and irinotecan are frequently used to target Top1 in ovarian, colon, and small cell lung cancer patients. Both topotecan and SN38, the active metabolite of irinotecan, stabilize Top1-DNA cleavage complexes (Top1cc), which are subsequently converted into DNA damage during DNA replication and transcription. The conversion of single stranded breaks (SSB) into double stranded breaks (DSB) during stalling of the replication machinery is the primary cytotoxic effect of Top1 poisons [2]. As a consequence, defects of tumor cells in proper repair of DSB provide an Achilles heel that can be targeted using Top1 inhibitors. An example is the increased topotecan sensitivity of cultured cells that are deficient in BRCA1 function [3], which is critical for error-free repair of DSB by homologous recombination (HR) [4].

We have previously studied topotecan responses in a genetically engineered mouse model for BRCA1-deficient breast cancer [5]. Despite high initial sensitivity, tumors were not eradicated and eventually all tumors acquired resistance to the maximum tolerable dose (MTD) of topotecan. About half of the tumors acquired resistance by overexpression of ABCG2 (or Breast Cancer Resistance Protein/BCRP). ABCG2 is an ATP-binding cassette (ABC) efflux transporter [6]–[8], and its overexpression in cultured cells was found to cause resistance to various clinically used anti-cancer drugs, such as topotecan and irinotecan [2], [9]. In our mouse model, we found that tumor-specific ablation of this efflux transporter substantially increased overall survival of tumor-bearing animals [5]. This observation unambiguously confirmed that induction of ABCG2 expression is an effective mechanism of mammary tumors to evade topotecan-induced DNA damage.

At present, useful *in vivo* strategies to reverse ABCG2-mediated drug resistance in patients are lacking. ABCB1/P-gp inhibitors like elacridar or tariquidar also inhibit ABCG2 to some extent [10], [11]. However, thus far the clinical benefit of these inhibitors is modest [12]–[14]. The mycotoxin fumitremorgin C (FTC) was identified as a more specific ABCG2 inhibitor [15], [16], but neurotoxicity compromised its clinical potential [17]. Nevertheless, less toxic FTC analogues were explored, and of these Ko143 was found to be the most potent and specific inhibitor, increasing oral topotecan availability in *Abcb1a/b* knockout mice 4–6-fold [17].

A complication of the systemic application of ABCG2 inhibitors is the fact that ABCG2 is expressed in normal tissues and protects them against xenotoxins [18]. In particular, ABCG2 contributes to the blood-brain barrier [19], and its expression in the liver, gut and kidney results in increased drug clearance. Hence, when combining an ABCG2 inhibitor with topotecan, it may be difficult to distinguish tumor-specific ABCG2 inhibition versus increased drug exposure due to reduced excretion. To be able to make this distinction, the use of ABCG2-deficient mice is helpful. ABCG2-proficient tumors can be grafted into syngeneic mice that lack ABCG2. This allows the analysis of tumor cell-specific effects of the inhibitor. We have shown that the spontaneous mammary tumors of our BRCA1 model can be transplanted orthotopically into syngeneic mice without loss of their genomic profile, morphology, or sensitivity to drug [5], [20], [21]. By transplanting ABCG2-proficient *Brca1^−/−^;p53^−/−^* mammary tumors derived from FVB/N mice into ABCG2-deficient hosts of the same strain, we show here that Ko143 is indeed useful for reversing ABCG2-mediated topotecan resistance *in vivo*. Nevertheless, the benefit is modest and Ko143 does not result in a clearly detectable increase of intratumoral topotecan concentration. As an alternative approach to overcome ABCG2-mediated resistance, we therefore tested a polyethylene glycol-conjugated (‘pegylated’) Top1 inhibitor EZN-2208, a SN38 conjugate. In xenografts EZN-2208 results in higher and longer-lasting exposure of tumors to the irinotecan metabolite SN38 compared with irinotecan itself [22]–[24]. In our model, we found that EZN-2208 results in durable responses of ABCG2-expressing BRCA1-deficient mammary tumors, in contrast to topotecan or irinotecan treatment.

## Materials and Methods

### Spontaneous Mammary Tumors, Orthotopic Transplantations, Recipient Animals and Drug Intervention Regimens

All animal experimental procedures have been conducted according to the standard operating procedures of the lab animal facility and were approved by the Animal Ethics Committee of The Netherlands Cancer Institute under references 08.001-B44, 08.001-B45, 11.006-B06 and 11.006-B10.

**Figure 1:**
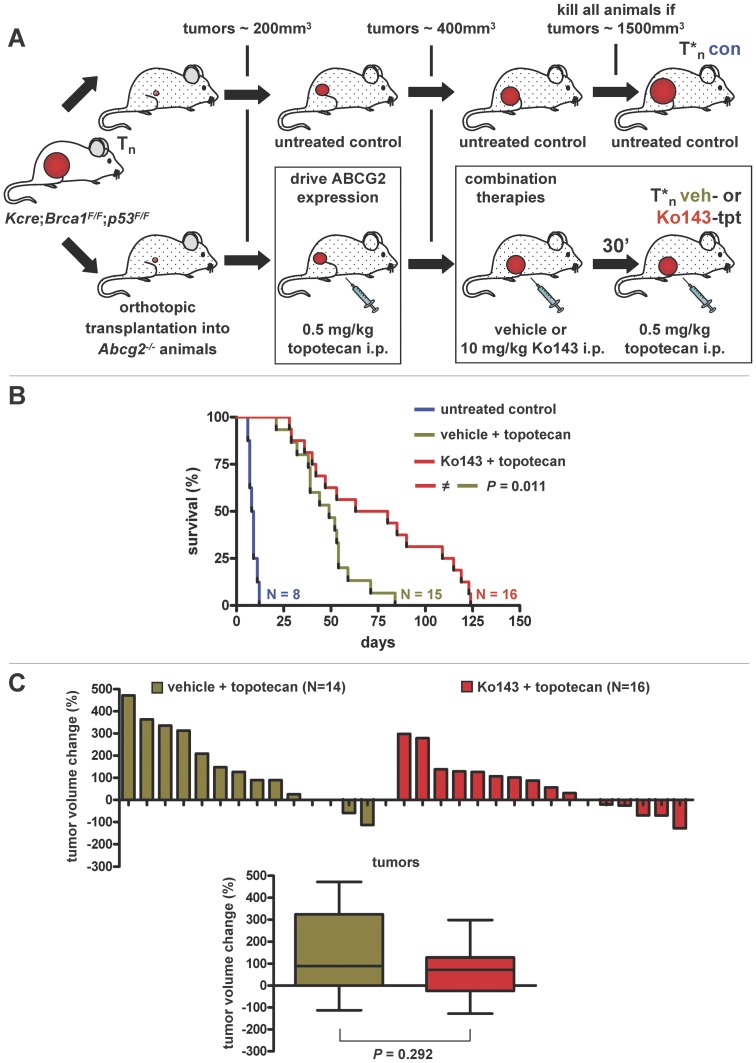
Efficacy of Ko143+ topotecan combination therapy in *Brca1^−/−^*;*p53^−/−^* mammary tumors. A, Experimental outline. Spontaneous mammary tumors from *K14cre;Brca1^F/F^;p53^F/F^* females were orthotopically transplanted into six- to eight-week-old *Abcg2^−/−^* syngeneic recipients. When tumors reached a volume of about 200 mm^3^, animals were either left untreated (control) or injected i.p. with 0.5 mg topotecan per kg body weight on days 0–4 and 14–18 resulting in elevated ABCG2 expression. When tumors doubled in size (∼400 mm^3^), animals were injected i.p. with either vehicle +0.5 mg topotecan or 10 mg Ko143+0.5 mg topotecan combination therapy per kg body weight on days 0–4 and 14–18. If the tumor volume shrank to less than 50% of the start volume, treatments were stopped until tumors relapsed to 100%. The experiment was terminated when tumors reached a volume of about 1500 mm^3^. B, Kaplan-Meier (K-M) survival curves of untreated and combination therapy-treated tumor-bearing animals. Eight individual tumors were tested, and per tumor one untreated control (blue line), two vehicle + topotecan- (green line) and two Ko143+ topotecan-treated animals (red line) were included. For the vehicle + topotecan group one tumor did not grow out after transplantation. The *P* value was calculated using the Log-rank test. C, Waterfall plots (upper panel) showing tumor volume change (%) after 5 days of combination therapy per individual mouse, with relative volumes normalized to the treatment start volume (i.e. about 200 mm^3^). Box and whiskers plots (lower panel) summarizing the waterfall plot data. Lines represent the median response, while the whiskers show the maximum and minimum values. The *P* values was calculated using the Mann Whitney test.

The generation, genotyping, orthotopic transplantation, daily animal weight and mammary tumor caliper measurements as well as the sampling of *Brca1^−/−^*;*p53^−/−^* mammary tumors were performed as described previously [5], [25]. Six- to eight-week-old FVB/N recipient animals were purchased from Harlan, while *Abcg2^−/−^* host animals of the same age and genetic background were bred within our lab animal facility [26]. The experimental outline of the Ko143 (or vehicle) + topotecan combination therapy interventions in *Abcg2^−/−^* tumor-bearing animals is described under the results (Fig. 1A). Intervention with topotecan, irinotecan and EZN-2208 in wildtype FVB/N tumor-bearing animals started when a tumor volume of about 200 mm^3^ was reached. Animals were either left untreated (control) or received 4 mg topotecan, 40 mg irinotecan or 10 mg EZN-2208 (SN38 equivalents) per kg body weight as a regimen of five consecutive i.v. injections on days 0, 2, 4, 6 and 8. When a tumor volume of about 1500 mm^3^ was reached, animals were killed by CO_2_ and tumor samples were harvested for further analyzes.

### Drug Injection Solutions

Ko143 was purchased from Tocris Bioscience (Minneapolis, MN, USA) and used by diluting 10 mg/mL DMSO stocks in 15% (w/v) 2-hydroxyl-propyl-β-cyclodextrine/PBS to a final volume of 1 mg/mL and administered at 10 µL/g of body weight by i.p. injection. Topotecan was provided by GlaxoSmithKline PLC (London, UK) and dissolved in 5% (w/v) glucose to yield a solution of 0.4 mg/mL (of active compound) and administered at 10 µL/g of body weight by i.p. injection. EZN-2208 was provided by ENZON pharmaceuticals Inc. (Piscataway, NJ, USA) and dissolved in saline to yield a solution of 1 mg/mL (SN38 equivalents) and administered at 10 µL/g of body weight by i.v. injection. Irinotecan was purchased from Pfizer Inc. (New York, NY, USA) and used by diluting 20 mg/mL stocks in saline to yield a solution of 4 mg/mL and administered at 10 µL/g of body weight by i.v. injection.

### Immunohistochemical Analysis of ABCG2

Immunohistochemical stainings were performed as described previously [5]. Briefly, ABCG2 was probed with the rat anti-mouse monoclonal (BXP53) from Abcam (ab24115, 1∶400) and detected with a biotinylated rabbit anti-rat secondary antibody (Dakocytomation, E0468, 1∶100).

### Collection of Pharmacokinetics Samples and Quantification of Topotecan Levels

While animals were under isoflurane anesthesia, whole blood was collected by cardiac puncture and transferred into heparinized tubes on ice. Next, the animals were killed by cervical dislocation and their tumors dissected. Blood was centrifuged at 4000 rpm and 4°C for 5 min to separate the plasma fraction. After thawing at 4°C, the mouse tumors were weighed and homogenized in 1% (w/v) BSA in water (equivalent to 500 mg tumor in 2.5 mL volume), using a FastPrep-24 high speed bench top homogenizer (MP-Biomedicals, Santa Ana, CA, USA) at 6.0 M/s for 30 s in 4.5 mL tubes. Homogenized tumor and plasma samples were stored at −20°C until sample pretreatment for reversed-phase high performance liquid chromatography (RP-HPLC) analysis. Total topotecan levels (lactone plus carboxylate form) were determined by using a validated RP-HPLC method, as described previously [27].

## Results

### Efficacy of Ko143+ Topotecan Combination Therapy in *Abcg2^−/−^* Tumor-bearing Animals

To study the effect of ABCG2 inhibition on topotecan efficacy in a tumor-specific fashion, we transplanted spontaneous ABCG2-proficient *Brca1^−/−^;p53^−/−^* mammary carcinomas [25], derived on a FVB/N background, orthotopically into ABCG2-deficient recipients [26] of the same mouse strain (Fig. 1A). Since ABCG2 expression in normal tissues is known to contribute to topotecan clearance [19], we had to lower the topotecan dose in *Abcg2^−/−^* mice substantially (Fig. S1A). In fact, the topotecan MTD of these animals was 4-fold lower than in wild-type animals (0.5 mg instead of 2 mg i.p. per kg body weight on days 0–4 and 14–18). The combination of this topotecan dose with 10 mg Ko143 i.p. per kg body weight on days 0–4 and 14–18 was still tolerable in mice that lack ABCG2 (Fig. S1B). This supports the notion that Ko143 is an ABCG2-specific inhibitor which does not cause serious off-target complications.

**Figure 2:**
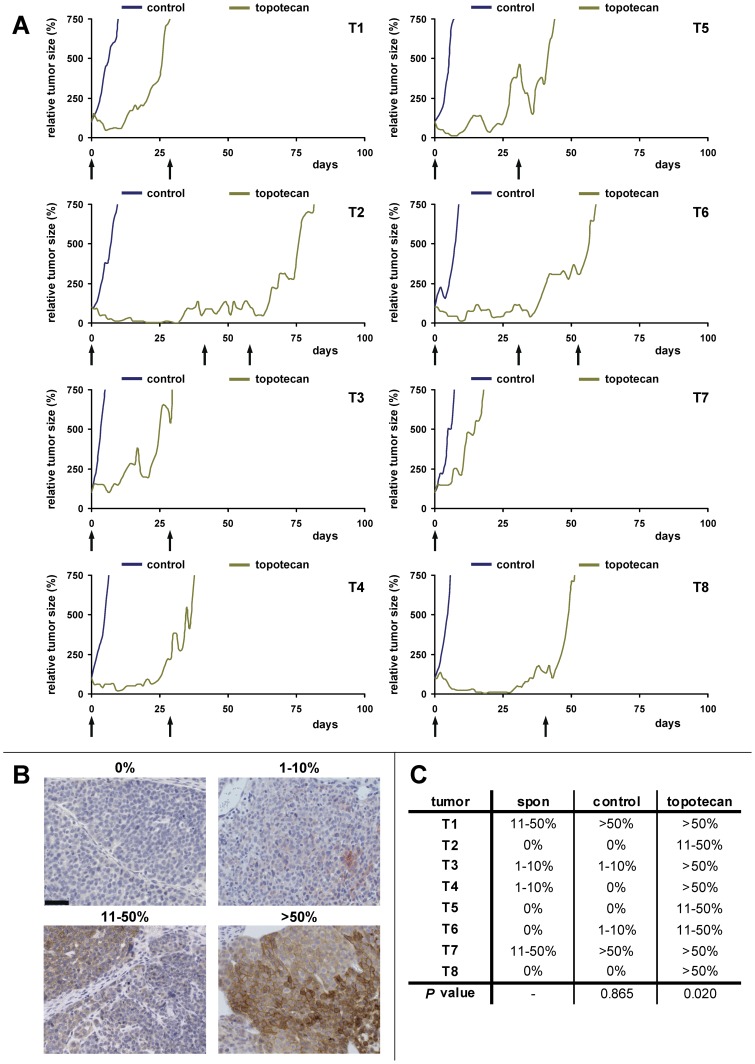
Topotecan response and ABCG2 immunoreactivity of eight individual *Brca1^−/−^*;*p53^−/−^* mammary tumors. A, Relative tumor volume (%) of matched control (blue lines) and topotecan-treated (green lines) tumors over time. Each arrow indicates one dosing regimen of i.p. injections of 0.5 mg topotecan per kg body weight on days 0–4 and 14–18. B, Semi-quantification of ABCG2 immunoreactivity. Representative micrographs of four classes of stained tumor cells (0%, 1–10%, 11–50% and more than 50% of counted cells in 10 independent 400x magnification fields are ABCG2-positive) are shown. C, Table indicating ABCG2 immunoreactivity of the spontaneous (spon), untreated control (control) and topotecan-treated samples per individual tumor. *P* values of the spon - control (0.865) and spon - topotecan (0.020) comparisons were calculated using the Wilcoxon rank-sum test.

We then investigated the topotecan + Ko143 response of 8 randomly chosen ABCG2-proficient *Brca1^−/−^;p53^−/−^* mammary donor tumors in ABCG2-deficient hosts (Fig. 1A–C). When tumors reached a volume of 150–250 mm^3^ after transplantation, animals were either left untreated (control) or injected with topotecan until the tumor volume doubled to about 400 mm^3^ (Fig. 1A). This topotecan selection step was applied to drive ABCG2 expression in these naïve tumors following orthotopic transplantion, as we have reported previously [5]. As expected, there was a clear induction of ABCG2 in the topotecan-resistant tumors also in the donor tumors used for this study (Fig. 2 and Table S2). Once the tumors had doubled in size under topotecan selection, animals were either treated with vehicle + topotecan or Ko143+ topotecan combination therapy. Compared with vehicle + topotecan, Ko143+ topotecan combination therapy significantly (*P* = 0.022, Log-rank test) increased overall survival of the *Abcg2^−/−^* animals that carried *Brca1^−/−^;p53^−/−^* mammary tumors (Fig. 1B). Nevertheless, the median survival increased only from 52 to 71.5 days, showing that the benefit of adding Ko143 to topotecan therapy was rather modest. Indeed, when tumor volume changes after 5 days of therapy were plotted per individual mouse (Fig. 1C), no significant (*P* = 0.292, Mann Whitney test) increase in tumor shrinkage was found after addition of Ko143.

### Ko143 and Topotecan Pharmacokinetics

**Figure 3:**
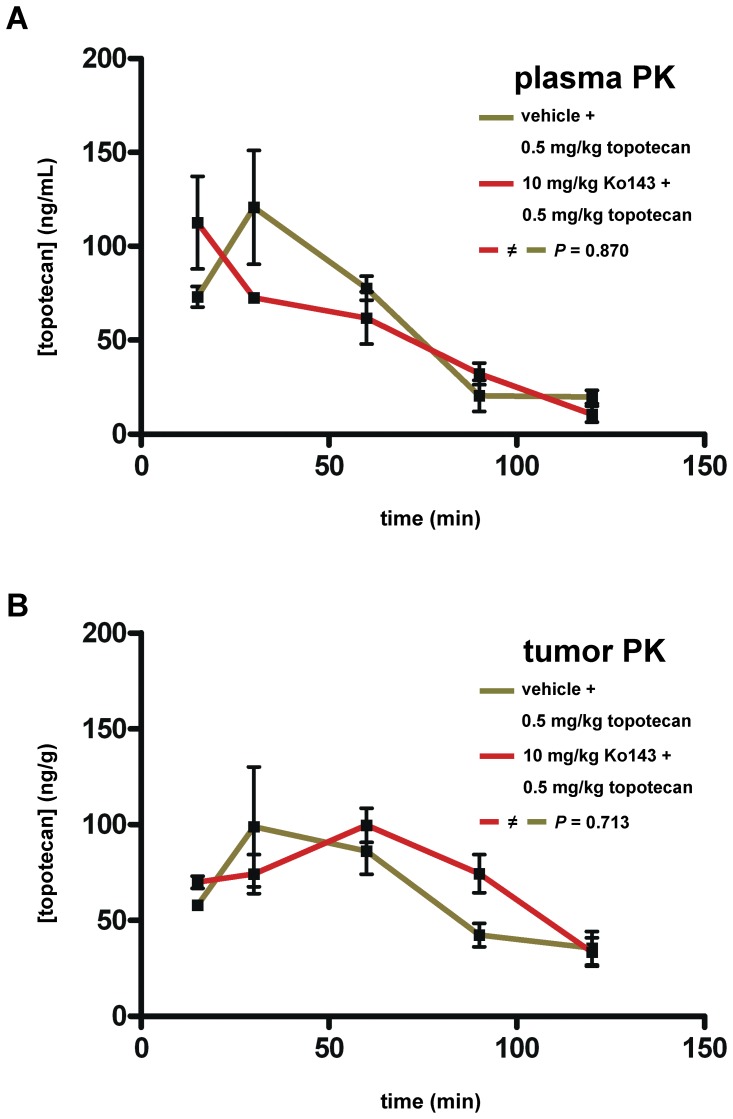
Plasma and tumor topotecan pharmacokinetics (PK). A, Plasma topotecan PK. *Abcg2^−/−^* animals, carrying ABCG2-positive tumor T1 (Fig. 2C), were treated with either vehicle +0.5 mg topotecan (green line) or 10 mg Ko143+0.5 mg topotecan (red line) per kg body weight and plasma was collected at 15, 30, 60, 90 and 120 minutes following i.p. injection. Error bars indicate standard deviations of the mean of at least 3 animals per time point. B, Tumor topotecan PK. The tumors of the animals in A were harvested and homogenized to determine topotecan concentrations at the same time points. Error bars indicate standard deviations of the mean of at least 3 animals per time point. *P* values were calculated using the unpaired t-test.

To quantify the effect of tumor-specific ABCG2 inhibition on topotecan accumulation, we developed a reverse phase high-performance liquid chromatography (RP-HPLC) assay to determine Ko143 concentrations in plasma and tumor matrix, using FTC as an internal standard (Zander *et al*., submitted for publication). Ko143 was administered to *Abcg2^−/−^* tumor-bearing animals by i.p. injections of 15% (w/v) 2-hydroxyl-propyl-β-cyclodextrine/PBS solutions of concentrated Ko143 DMSO stocks. To validate that this formulation yields effective Ko143 plasma and tumor levels, we performed a time course experiment. This confirmed that Ko143 reaches transplanted mammary tumors after i.p. injection, as reported elsewhere (Zander *et al*., submitted for publication). In contrast to *Abcb1a/b^−/−^* animals, in which Ko143 treatment resulted in a 4-fold increase in plasma topotecan concentration [17], in our *Abcg2^−/−^* tumor-bearing animals topotecan pharmacokinetics (PK) did not significantly (*P* = 0.870, unpaired t-test) differ between vehicle + topotecan- (Fig. 3A, green line) and Ko143+ topotecan-treated animals (Fig. 3A, red line). This suggests that Ko143 does not block other ABC transporters which may alter topotecan PK. This result is also consistent with the absence of increased toxicity when topotecan is combined with Ko143 (Fig. S1B).

Unexpectedly, when we measured topotecan pharmacokinetics in the ABCG2-positive tumor T1 (Fig. 2C) as described previously [27], we were unable to detect significant differences between the vehicle + topotecan-treated (Fig. 3B, green line) and Ko143+ topotecan-treated animals (Fig. 3B, red line) (*P* = 0.713, unpaired t-test). The short plasma half-life of Ko143 of about 1 hour (Zander *et al*., submitted for publication) may have contributed to this outcome. Most likely, the increase in topotecan levels within tumors after Ko143 treatment, which must have been responsible for the increased overall survival (Fig. 1B), was rather small. Hence, Ko143 treatment may not be the optimal strategy to increase intratumoral Top1 inhibitor levels.

### EZN-2208 as Alternative Strategy to Enhance Tumor-specific Drug Accumulation and Reverse ABCG2-mediated Resistance

Given this limited success of the ABCG2 inhibitor Ko143, we tested EZN-2208, a pegylated form of the active irinotecan metabolite SN38 [28], to treat ABCG2-positive *Brca1^−/−^;p53^−/−^* mammary tumors. Like topotecan, the topoisomerase I inhibitor SN38 is an ABCG2 substrate [29], [30], and therefore drug-naïve tumors with ABCG2-positive nests of tumor cells should quickly acquire resistance during irinotecan treatment. We became interested in EZN-2208, since pegylation promotes tumor-specific drug accumulation by exploiting the phenomenon known as the enhanced permeability and retention effect of solid tumors [31]. Sustained SN38 release from EZN-2208 in the tumor stroma may counteract cellular ABCG2-mediated efflux and maintain a steep diffusion gradient towards the topoisomerase I target within the tumor cell nuclei. Such prolonged tumor-specific drug delivery may help to overcome ABCG2-mediated resistance.

For our analysis, we compared EZN-2208 versus topotecan or irinotecan efficacy in drug-naïve *Brca1^−/−^;p53^−/−^* mammary tumors that showed ABCG2 expression in tumor cell subpopulations. In several spontaneous tumors (T1, T3, T4, T7) we observed varying amounts of ABCG2-positive nests of tumor cells (Fig. 2B+C). Obviously, these cells were selected during topotecan treatment, since there is a clear enrichment of ABCG2-positive cells in the resistant tumors (Fig. 2C). Moreover, these tumors acquire topotecan resistance more rapidly (Fig. 2A). In addition to T1 and T7, we found three other spontaneous tumors (named here T9, T10 and T11) with more than 10% ABCG2-positive tumors cells in our panel of 28 *Brca1^−/−^;p53^−/−^* mammary tumors derived from FVB/N mice (Zander *et al*., submitted for publication). Importantly, ABCG2-positive cells were still present after orthotopic transplantation of all 5 donor tumors (Table S1).

**Figure 4:**
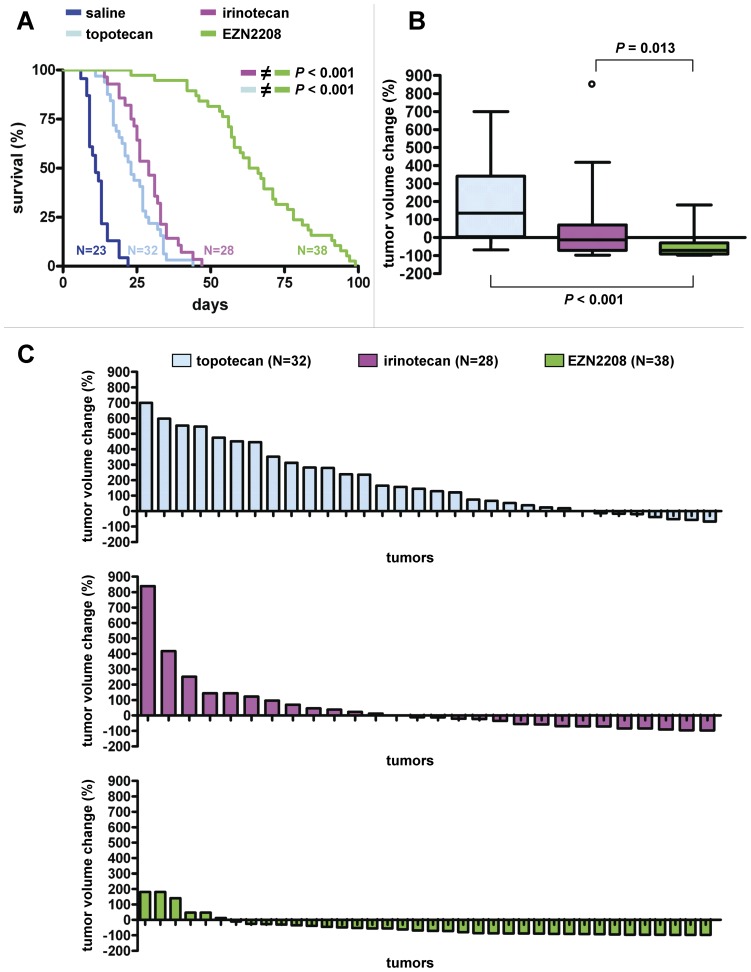
Efficacy of topotecan, irinotecan and EZN-2208 in five ABCG2-positive *Brca1^−/−^*;*p53^−/−^* mammary tumors. A, K–M curves showing survival (%) until a tumor volume of about 1500 mm^3^ was reached after one regimen of five consecutive i.v. injections on days 0, 2, 4, 6 and 8 of saline- (dark blue line, N = 23), topotecan- (light blue line, 4 mg i.v., N = 32), irinotecan- (pink line, 40 mg i.v., N = 28) and EZN-2208-treatments (green line, 10 mg SN38 equivalents i.v., N = 38) per kg body weight. *P* values were calculated using the Log-rank test. B, Box and whiskers plots indicating per drug the tumor volume change (%) after two weeks of treatment. Lines represent the median response, while the whiskers show the maximum and minimum values; the small circle is an outlier. *P* values were calculated using the Mann Whitney test. C, Waterfall plots showing tumor volume change (%) after two weeks of treatment per individual mouse, with relative volumes normalized to the treatment start volume.

Before drug interventions were started using these tumors, we established the maximum tolerable EZN-2208 dose in our mouse model, guided by earlier studies in xenograft models [23]. When EZN-2208 was injected i.v. at 10 mg (SN38 equivalents) per kg body weight on days 0, 2, 4, 6 and 8, the weight loss was still acceptable (about 10%, Fig. S2A). The MTD of topotecan and irinotecan was 4 mg and 40 mg per kg body weight, respectively, using this regimen of five consecutive i.v. injections (Fig. S2B). These doses were then given to mice carrying the orthotopically transplanted ABCG2-positive *Brca1^−/−^;p53^−/−^* mammary tumors T1, T7, T9, T10 and T11 (Fig. 4). Compared with both the irinotecan- (pink line) and topotecan-treated tumor-bearing animals (light blue line), we found a dramatic increase in survival until a tumor volume of about 1500 mm^3^ was reached after EZN-2208 treatment (Fig. 4A, green line, *P*<0.001, Log-rank test). This result was found for all five ABCG2-positive tumors individually (Fig. S3). Moreover, when tumor volume changes after two weeks of treatment were compared (Fig. 4B+C), EZN-2208 outperformed both irinotecan (*P* = 0.013, Mann Whitney test) and topotecan (*P*<0.001, Mann Whitney test). This was still the case for topotecan, but not for irinotecan when tumor volume changes were analysed per individual ABCG2-positive tumor (Fig. S4). Together, these data show that the use of EZN-2208 is an effective strategy to target ABCG2-positive *Brca1^−/−^;p53^−/−^* mammary tumors.

## Discussion

Using a genetically engineered mouse model for BRCA1-deficient breast cancer, we investigated therapeutic strategies to overcome ABCG2-mediated resistance to clinically used topoisomerase I inhibitors. We found that ABCG2-positive *Brca1^−/−^;p53^−/−^* mammary tumors are highly sensitive to EZN-2208, a pegylated SN38 compound. Compared with the MTD of irinotecan or topotecan, ABCG2-positive tumors shrank substantially in size and the time until relapse was greatly increased. Previously, EZN-2208 was also shown to be superior compared with irinotecan in xenograft models of neuroblastoma, B-cell non-Hodgkin’s lymphoma, breast, colon and pancreatic cancer [22]–[24]. Polyethylene glycol (PEG)-conjugation of the active irinotecan metabolite SN38 generates a water soluble camptothecin analogue [28] with enhanced pharmacokinetics profile as shown by Sapra *et al*. [23] in plasma and tumors of MX-1 breast cancer xenografted nude mice. Pegylation shields a conjugated compound from clearance by the liver reticulo-endothelial system and impairs glomerular filtration [32], [33], resulting in a considerably prolonged plasma half-life compared with irinotecan [23]. Our results suggest that EZN-2208 is capable of overcoming ABCG2-mediated drug resistance *in vivo*. This may be due to better drug delivery towards tumors by the enhanced permeability and retention (EPR) effect, as suggested previously [31], [34]. The EPR effect is based on the presence of fenestrated blood vessels in tumors, which allow pegylated molecules to enter the tissue. Lack of effective lymphatic drainage within tumors further increases local drug concentration. The poor penetration of pegylated drug into normal tissues may also explain the absence of increased toxicity despite higher and longer-lasting plasma levels [23]. Local esterase activity within the tumor cleaves off the bulky PEG moiety and releases SN38, which passes the plasma membrane and inhibits nuclear topoisomerase I. ABCG2 is apparently not a sufficiently effective defense mechanism against sustained SN38 delivery by EZN-2208. The PEG-liposome encapsulated formulation of the topoisomerase II inhibitor doxorubicin (Doxil or Caelyx) [35] also showed superior efficacy in preclinical mouse models compared with free doxorubicin [36], [37], and was also found to bypass P-glycoprotein-mediated drug efflux [38].

We show here that adding the specific ABCG2 inhibitor Ko143 to topotecan therapy increases the overall survival of animals carrying *Brca1^−/−^;p53^−/−^* mammary tumors. This is not unexpected, given our recent finding that the survival of topotecan-treated animals carrying ABCG2-deficient *Brca1^−/−^;p53^−/−^* mammary tumors is markedly increased over that of animals with ABCG2-proficient tumors. By using ABCG2-proficient tumors in ABCG2-deficient hosts, we ensured that the Ko143 effect was indeed tumor-specific and not due to drug clearance differences. Despite the increase in overall survival, the actual benefit of Ko143 was rather modest. Moreover, we failed to detect increased topotecan accumulation in these tumors. We have shown that dosing animals with 10 mg Ko143 per kg body weight resulted in tumor Ko143 levels of more than 250 ng/g for at least two hours following i.p. injection (Zander *et al*., submitted for publication). Such levels are well above the EC_90_ concentration of 25 nM as determined by Allen *et al*. [17] in tumor cell cultures with *Abcg2* amplifications [17], [39], [40]. ABCG2 inhibition by Ko143 in the mouse tumors should therefore be effective during at least this period following administration. However, the differences in tumor topotecan levels were probably small and our RP-HPLC quantification method did not detect them, given the variation between individual animals.

Since the initial report of Ko143 in the literature, several new inhibitors of ABCG2 have been identified [41]. PZ-39, for example, seems to be an attractive candidate, not only inhibiting ABCG2, but also accelerating its lysosome-dependent degradation [42], [43]. This two-pronged action may disrupt tumor ABCG2 function in a more profound and longer-lasting way than treatment with Ko143. However, these newer ABCG2 inhibitors are still at an early stage of development and, in contrast to Ko143, toxicity data are lacking.

Since ABC transporters have been shown to play a critical role in chemoresistance of cultured tumor cells, it has been a long-standing goal to use inhibitors of these transporters as a strategy to overcome resistance in cancer patients [44]. However, successful clinical application of combination therapies has proven to be difficult, often only increasing toxicity of the chemotherapeutic agent without actually improving its efficacy [12], [13], [45]. Since ABC transporters are also involved in the disposition of most of these drugs, pharmacokinetic interactions may considerably complicate successful application of this therapy strategy in the clinic. To avoid intolerable toxicities, dosing of the cytostatic agents has often to be lowered in such combinations. Indeed, we also found that the topotecan MTD was 4-fold lower in *Abcg2^−/−^* tumor-bearing animals compared with wild-type animals.

Given these disadvantages, EZN-2208 may be a helpful alternative to the use of ABCG2 inhibitors in combination with topoisomerase inhibitors. Moreover, improved drug delivery to tumors without increased toxicity in other tissues is attractive in combination with other therapeutics that target BRCA-deficient cancer, such as the PARP inhibitor olaparib (AZD2281) [21]. Previously, we treated ABCG2-deficient mammary tumors with topotecan-olaparib combination therapy and observed improved tumor response compared with topotecan monotherapy [5]. However, the topotecan dose had to be lowered 8-fold in this combination, because of the intestinal toxicity. Indeed, dose-limiting haematological toxicity was also reported for patients with advanced solid tumors in phase I clinical trials of the topotecan-olaparib and -ABT-888 combinations [46], [47]. Another reason for dose reduction of irinotecan are glucuronidation-impairing UGT1A1 polymorphisms, such as the UGT1A1*28 genotype, which is known to increase the risk of diarrhea [48]. Mild intestinal toxicity was observed in phase I and II trails with EZN-2208, but this was not dose-limiting [49], [50]. Combination of olaparib with EZN-2208 may therefore be tolerated better, potentially achieving tumor eradication by dose escalation.

In summary, two different therapeutic strategies were tested to overcome ABCG2-mediated topotecan resistance in *Brca1^−/−^*;*p53^−/−^* mouse mammary tumors. Although tumor-specific ABCG2 inhibition by Ko143 resulted in improved overall survival of tumor-bearing animals, the benefit was rather modest. In contrast, ABCG2-expressing tumors are highly sensitive to EZN-2208, resulting in a substantial increase in survival. Application of this pegylated SN38 formulation may therefore be a useful clinical approach to bypass ABCG2-mediated resistance to conventional camptothecin analogues in cancers with defects in homology-directed DNA repair.

## Supporting Information

Figure S1 **Relative animal weights in response to topotecan mono- or Ko143+ topotecan combination therapy.** A, Six- to eight-week-old *Abcg2^−/−^* females were injected i.p. with either 1 (red line), 0.75 (green line) or 0.5 mg topotecan (blue line) per kg body weight on days 0–4 and 14–18 (arrows) and weighed daily for 28 days. The average relative weight (%) of five animals per treatment is plotted and error bars indicate standard deviations. If weight loss approached 20%, animals were killed by CO_2_. B, Six- to eight-week-old *Abcg2^−/−^* females were injected i.p. with 10 mg Ko143 and 0.5 mg topotecan per kg body weight on days 0–4 and 14–18 (arrows). There was a 30 minute interval between Ko143 and topotecan i.p. injections. The average relative weight (%) of five animals is plotted and error bars indicate standard deviations.
(TIFF)

Figure S2 **Relative animal weights in response to EZN-2208, irinotecan and topotecan therapy.** A, Six- to eight-week-old FVB/N females were treated with one regimen of five consecutive i.v. injections on days 0, 2, 4, 6 and 8 of either saline (black line) or 15 mg (red line) and 10 mg (SN38 equivalents) EZN-2208 (green line) per kg body weight as indicated by the arrows and weighed daily for 28 days. The average relative weight (%) of five animals per treatment is plotted and error bars indicate standard deviations. If weight loss approached 20%, animals were killed by CO_2_. B, Six- to eight-week-old FVB/N females were treated with one regimen of five consecutive i.v. injections on days 0, 2, 4, 6 and 8 of either saline (black line) or 4 mg topotecan (light blue line) and 40 mg irinotecan (pink line) per kg body weight as indicated by the arrows. The average relative weight (%) of five animals per treatment is plotted and error bars indicate standard deviations.
(TIFF)

Figure S3 **Survival of topotecan-, irinotecan- and EZN-2208-treated animals per individual ABCG2-positive donor tumor.** K-M curves showing survival (%) until a tumor volume of about 1500 mm^3^ was reached after one regimen of five consecutive i.v. injections on days 0, 2, 4, 6 and 8 of saline- (dark blue lines), topotecan- (light blue lines, 4 mg i.v.), irinotecan- (pink line, 40 mg i.v.) and EZN-2208-treatments (green line, 10 mg SN38 equivalents i.v.) per kg body weight. The number of experimental animals per treatment are indicated next to each K-M curve. *P* values were calculated using the Log-rank test.
(TIFF)

Figure S4 **Topotecan, irinotecan and EZN-2208 response per individual ABCG2-positive donor tumor.** Waterfall plots showing tumor volume change (%) after two weeks of treatments per individual mouse, with relative volumes normalized to the treatment start volume. Box and whiskers plots summarizing the waterfall plot data. Lines represent the median response, while the whiskers show the maximum and minimum values; the small circles are outliers. *P* values were calculated using the Mann Whitney test.
(TIFF)

Table S1 ABCG2 immunoreactivity of EZN-2208 intervention tumors. In addition to the two ABCG2-positive tumors of the Ko143+ topotecan intervention study (Fig. 2C, T1 and T7), three additional spontaneous (spon) *Brca1^−/−^*;*p53^−/−^* mammary tumors (T9, T10 and T11) were selected with high ABCG2 expression and orthotopically transplanted into wild-type recipients to test EZN-2208 efficacy. Semi-quantified ABCG2 immunoreactivity of the untreated controls is indicated per individual donor tumor.
(TIFF)

Table S2 ABCG2 immunoreactivity of Ko143 intervention tumors. Semi-quantified ABCG2 immunoreactivity of the untreated controls, vehicle + topotecan- and Ko143+ topotecan-treated animals is indicated per individual donor tumor.
(TIF)
