# Supplementary figures and images for: EZN-2208 (PEG-SN38) Overcomes ABCG2-Mediated Topotecan Resistance in BRCA1-Deficient Mouse Mammary Tumors
Source: PLoS One. 2012 Sep 17;7(9):e45248. doi: 10.1371/journal.pone.0045248 (PMC3444454; doi:10.1371/journal.pone.0045248)

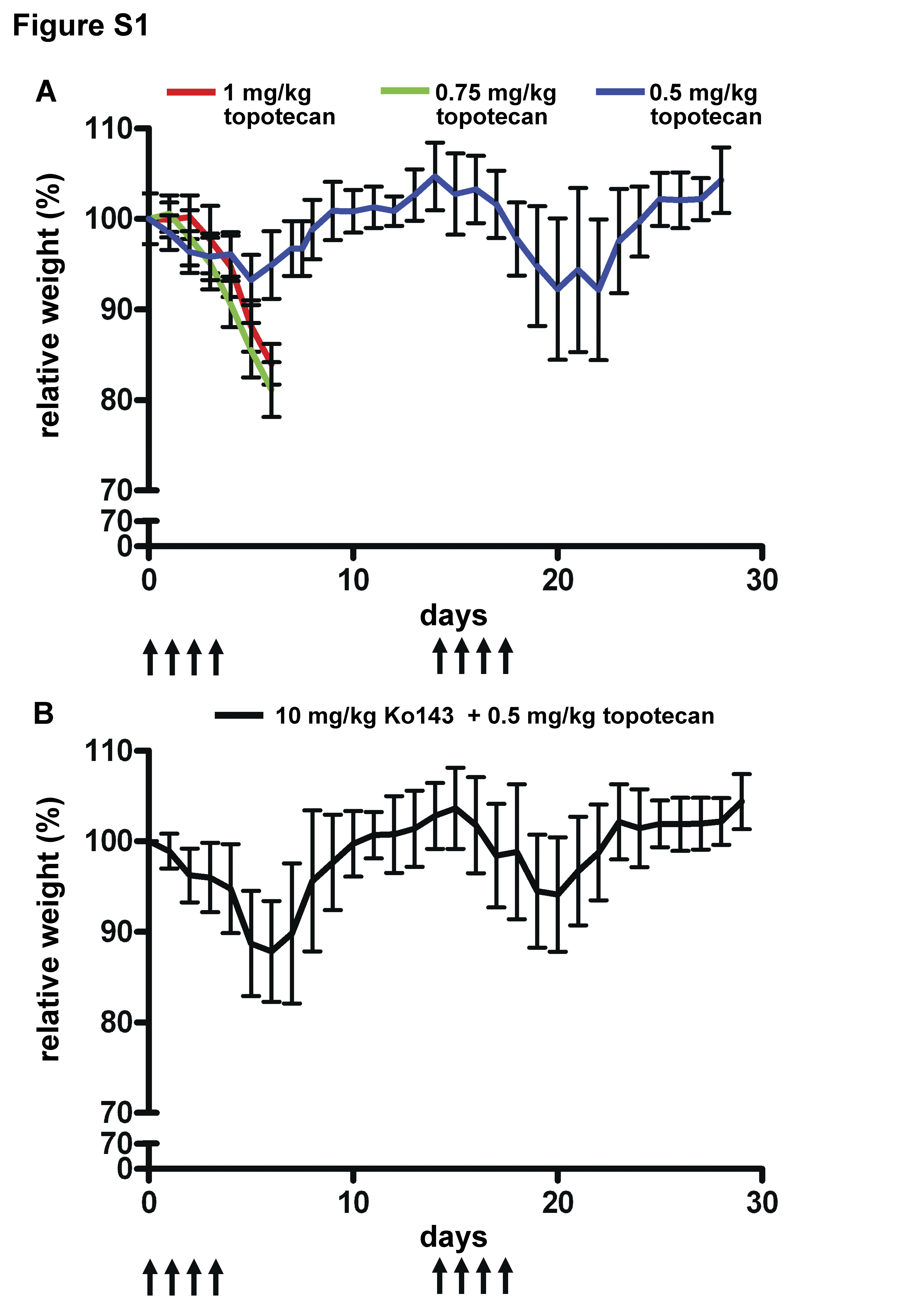

Supplement: Figure S1 — Relative animal weights in response to topotecan mono- or Ko143+ topotecan combination therapy. A, Six- to eight-week-old Abcg2−/− females were injected i.p. with either 1 (red line), 0.75 (green line) or 0.5 mg topotecan (blue line) per kg body weight on days 0–4 and 14–18 (arrows) and weighed daily for 28 days. The average relative weight (%) of five animals per treatment is plotted and error bars indicate standard deviations. If weight loss approached 20%, animals were killed by CO2. B, Six- to eight-week-old Abcg2−/− females were injected i.p. with 10 mg Ko143 and 0.5 mg topotecan per kg body weight on days 0–4 and 14–18 (arrows). There was a 30 minute interval between Ko143 and topotecan i.p. injections. The average relative weight (%) of five animals is plotted and error bars indicate standard deviations. (TIFF) [file pone.0045248.s001.tiff]

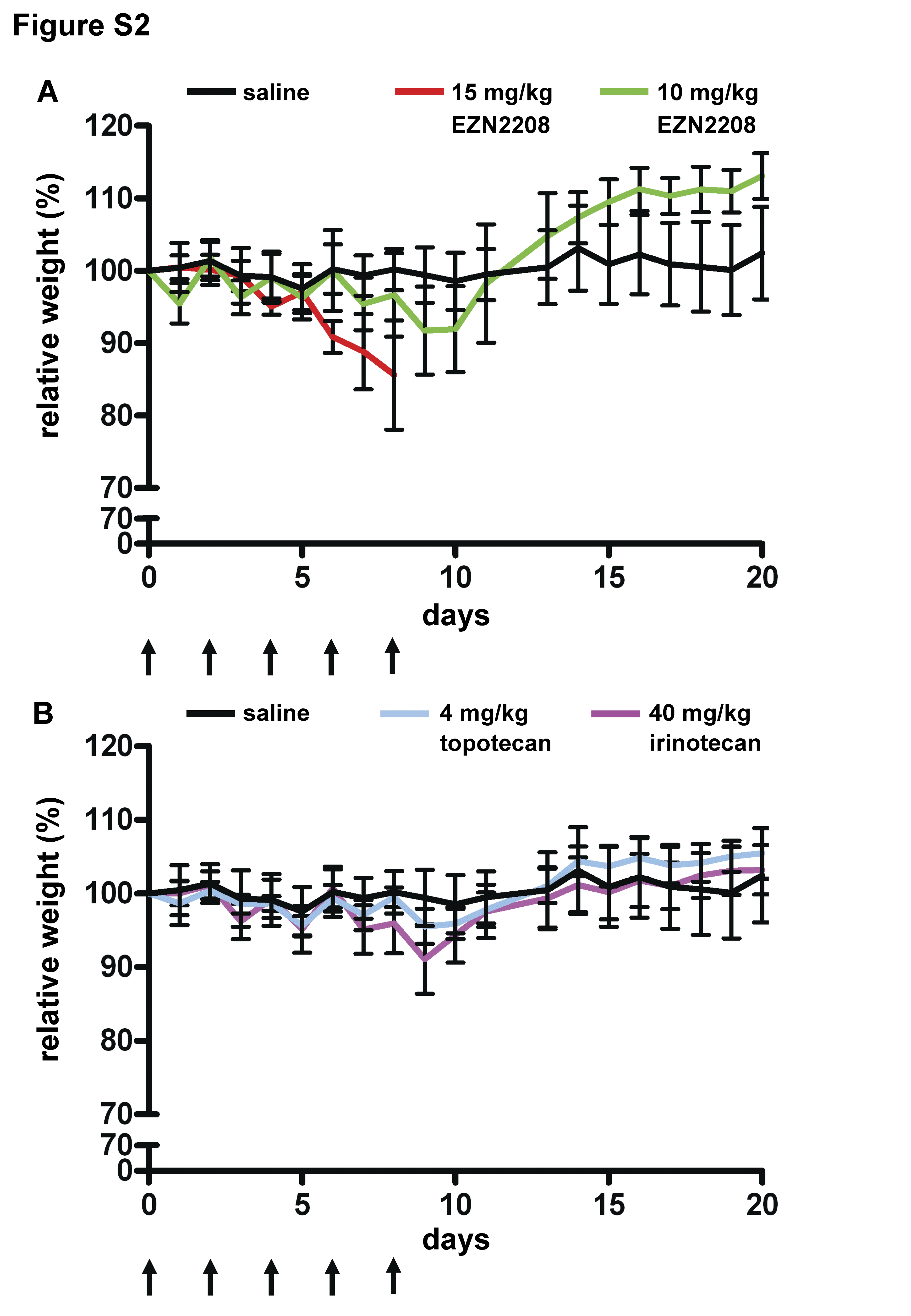

Supplement: Figure S2 — Relative animal weights in response to EZN-2208, irinotecan and topotecan therapy. A, Six- to eight-week-old FVB/N females were treated with one regimen of five consecutive i.v. injections on days 0, 2, 4, 6 and 8 of either saline (black line) or 15 mg (red line) and 10 mg (SN38 equivalents) EZN-2208 (green line) per kg body weight as indicated by the arrows and weighed daily for 28 days. The average relative weight (%) of five animals per treatment is plotted and error bars indicate standard deviations. If weight loss approached 20%, animals were killed by CO2. B, Six- to eight-week-old FVB/N females were treated with one regimen of five consecutive i.v. injections on days 0, 2, 4, 6 and 8 of either saline (black line) or 4 mg topotecan (light blue line) and 40 mg irinotecan (pink line) per kg body weight as indicated by the arrows. The average relative weight (%) of five animals per treatment is plotted and error bars indicate standard deviations. (TIFF) [file pone.0045248.s002.tiff]

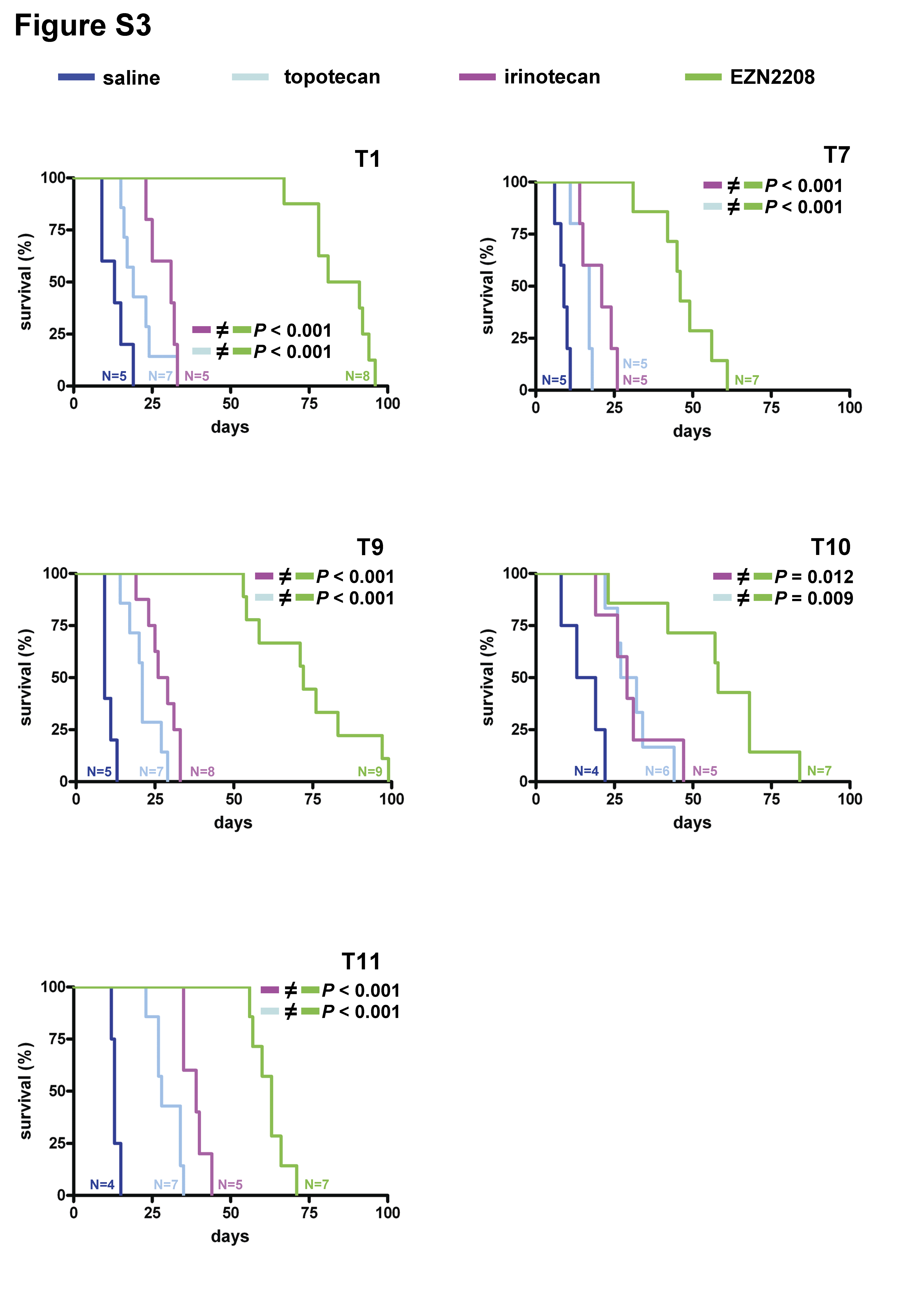

Supplement: Figure S3 — Survival of topotecan-, irinotecan- and EZN-2208-treated animals per individual ABCG2-positive donor tumor. K-M curves showing survival (%) until a tumor volume of about 1500 mm3 was reached after one regimen of five consecutive i.v. injections on days 0, 2, 4, 6 and 8 of saline- (dark blue lines), topotecan- (light blue lines, 4 mg i.v.), irinotecan- (pink line, 40 mg i.v.) and EZN-2208-treatments (green line, 10 mg SN38 equivalents i.v.) per kg body weight. The number of experimental animals per treatment are indicated next to each K-M curve. P values were calculated using the Log-rank test. (TIFF) [file pone.0045248.s003.tiff]

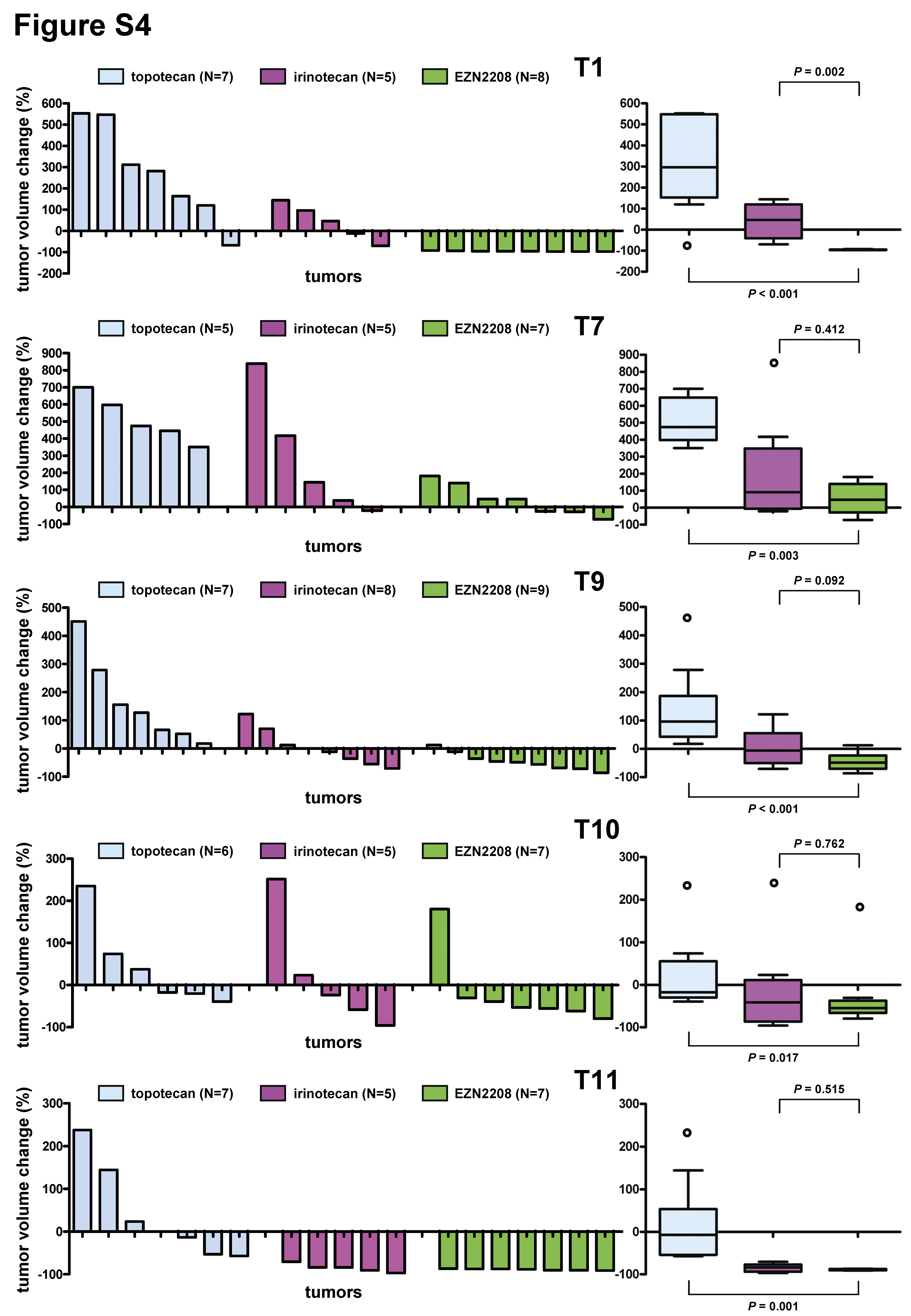

Supplement: Figure S4 — Topotecan, irinotecan and EZN-2208 response per individual ABCG2-positive donor tumor. Waterfall plots showing tumor volume change (%) after two weeks of treatments per individual mouse, with relative volumes normalized to the treatment start volume. Box and whiskers plots summarizing the waterfall plot data. Lines represent the median response, while the whiskers show the maximum and minimum values; the small circles are outliers. P values were calculated using the Mann Whitney test. (TIFF) [file pone.0045248.s004.tiff]

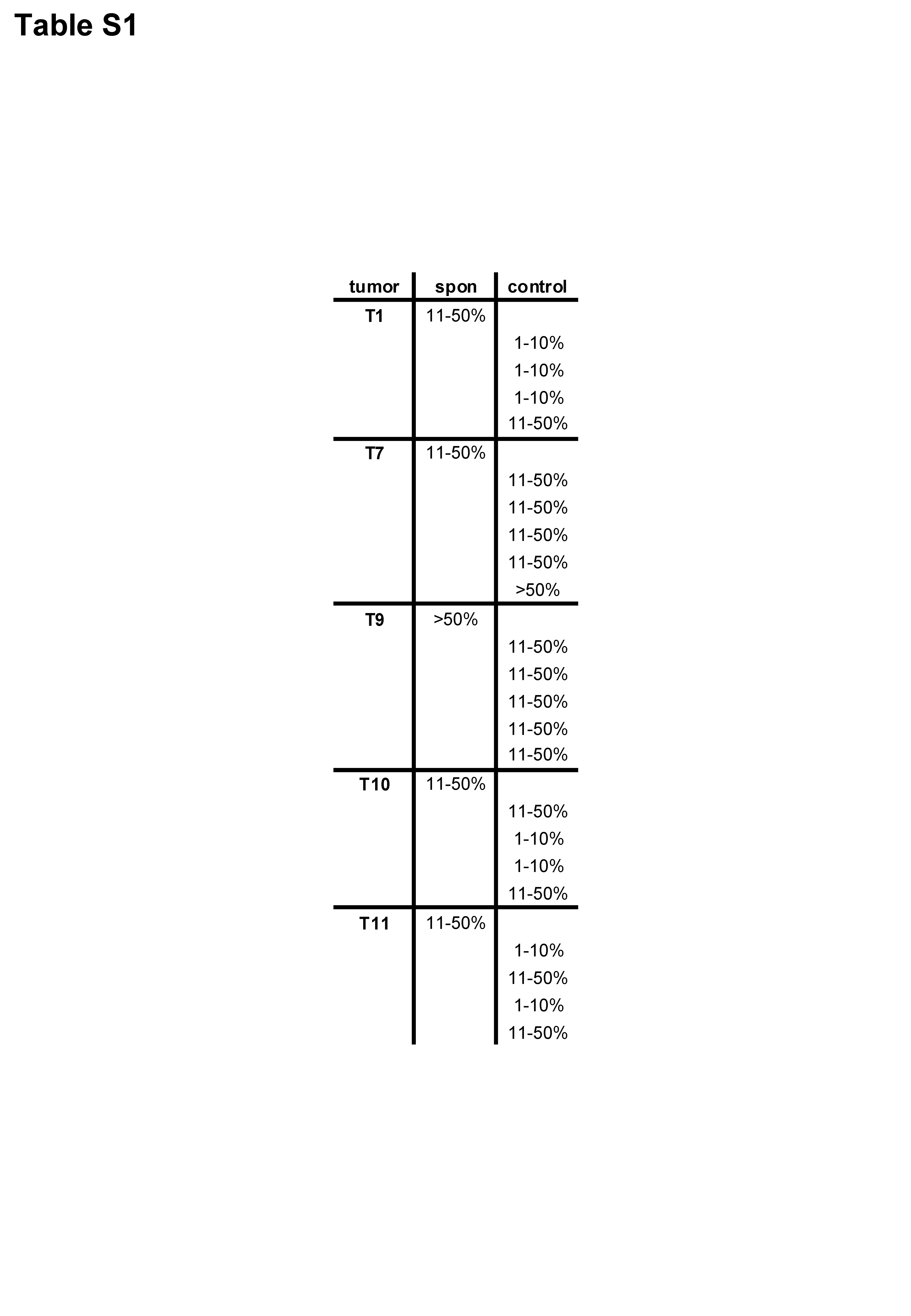

Supplement: Table S1 — ABCG2 immunoreactivity of EZN-2208 intervention tumors. In addition to the two ABCG2-positive tumors of the Ko143+ topotecan intervention study (Fig. 2C, T1 and T7), three additional spontaneous (spon) Brca1−/−;p53−/− mammary tumors (T9, T10 and T11) were selected with high ABCG2 expression and orthotopically transplanted into wild-type recipients to test EZN-2208 efficacy. Semi-quantified ABCG2 immunoreactivity of the untreated controls is indicated per individual donor tumor. (TIFF) [file pone.0045248.s005.tiff]

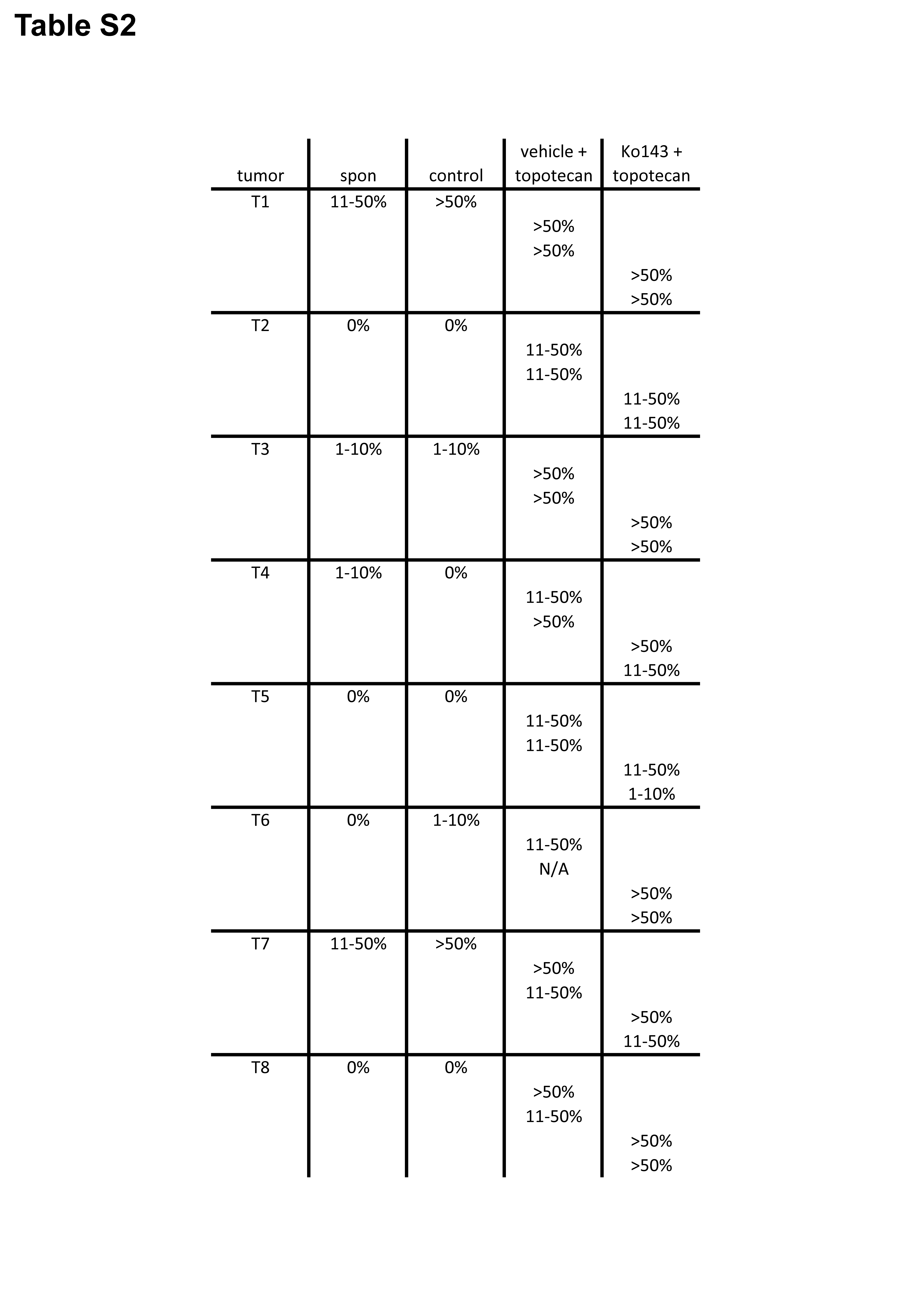

Supplement: Table S2 — ABCG2 immunoreactivity of Ko143 intervention tumors. Semi-quantified ABCG2 immunoreactivity of the untreated controls, vehicle + topotecan- and Ko143+ topotecan-treated animals is indicated per individual donor tumor. (TIF) [file pone.0045248.s006.tif]
